# EEG may serve as a biomarker in Huntington’s disease using machine learning automatic classification

**DOI:** 10.1038/s41598-018-34269-y

**Published:** 2018-10-31

**Authors:** Omar F. F. Odish, Kristinn Johnsen, Paul van Someren, Raymund A. C. Roos, J. Gert van Dijk

**Affiliations:** 1 Department of Neurology, University Medical Center Groningen, Groningen, The Netherlands; 2 MentisCura ehf., Reykjavík, Iceland; 3 Department of Neurology, Leiden University Medical Center, Leiden, The Netherlands

## Abstract

Reliable markers measuring disease progression in Huntington’s disease (HD), before and after disease manifestation, may guide a therapy aimed at slowing or halting disease progression. Quantitative electroencephalography (qEEG) may provide a quantification method for possible (sub)cortical dysfunction occurring prior to or concomitant with motor or cognitive disturbances observed in HD. In this pilot study we construct an automatic classifier distinguishing healthy controls from HD gene carriers using qEEG and derive qEEG features that correlate with clinical markers known to change with disease progression in HD, with the aim of exploring biomarker potential. We included twenty-six HD gene carriers (49.7 ± 8.5 years) and 25 healthy controls (52.7 ± 8.7 years). EEG was recorded for three minutes with subjects at rest. An EEG index was created by applying statistical pattern recognition to a large set of EEG features, which was subsequently tested using 10-fold cross-validation. The index resulted in a continuous variable ranging from 0 to 1: a low value indicating a state close to normal and a high value pointing to HD. qEEG features that correlate specifically with commonly used clinical markers in HD research were derived. The classification index had a specificity of 83%, a sensitivity of 83% and an accuracy of 83%. The area under the curve of the receiver operator characteristic curve was 0.9. qEEG analysis on subsets of electrophysiological features resulted in two highly significant correlations with clinical scores. The results of this pilot study suggest that qEEG may serve as a biomarker in HD. The indices correlating with modalities changing with the progression of the disease may lead to tools based on qEEG that help monitor efficacy in intervention studies.

## Introduction

Huntington’s disease (HD) is an autosomal dominant neurodegenerative disorder characterized by motor, cognitive and psychiatric symptoms with a mean age at onset between 30–50 years^1^. It is caused by an expanded cytosine-adenine-guanine (CAG) trinucleotide repeat in the huntingtin gene on the short arm of chromosome 4. The disease causes widespread brain pathology. Magnetic resonance imaging (MRI) studies in HD have revealed extensive brain atrophy, most notably in the striatum^2–4^. With disease progression, neurodegenerative changes further extend to the cortical grey-matter areas^5,6^. Cortical atrophy is found in both premanifest (preHD) as well as manifest stages of HD, with an increasing cortical thinning detectable with progressing disease severity^2,7^.

A challenge in HD research is to establish reliable markers to measure disease progression, both before and after disease manifestation, in preparation for the advent of new therapy aiming to slow or halt disease progression. This will be of tantamount importance for carriers of CAG repeat lengths of 40 or higher as they will develop manifest HD with certainty.

Electroencephalography (EEG) is an easy, cheap and rapid technique to assess (sub)cortical pathology. Quantitative electroencephalography (qEEG) provides objective parameters to assess (sub)cortical dysfunction occurring prior to or concomitant with motor or cognitive disturbances in HD. Combining such measures with clinical tests in HD gene carriers may provide added insights into progression of pathology and increased sensitivity for detecting subtle changes. Previous studies have found EEG abnormalities in HD^8^. A study using a different automated method compared to the one used in this paper, called automated artificial neural networks (ANN), showed promising results in discriminating between EEG’s of HD gene carriers and controls^9^.

In this pilot study, we hypothesized that machine learning automatic classification of EEG patterns may discern healthy controls from HD gene carriers. If so, this would be the first step to assess this technique as a longitudinal biomarker in HD. Secondly, we aimed to derive qEEG features that correlate with commonly used clinical and cognitive markers in HD research, known to change with disease progression. This is done to evaluate the usefulness of these qEEG features as biomarkers for tracking disease state and progression in HD.

## Materials and Methods

### Participants

Twenty-six HD gene carriers and 25 healthy controls were recruited from the Neurology outpatient clinic of the Leiden University Medical Center (LUMC), the Netherlands (Table 1). The preHD group (6 subjects) had a CAG repeat ≥ 40 with a total motor score on the Unified Huntington’s Disease Rating Scale (UHDRS-TMS) ≤ five. The early manifest HD group (20 subjects) had a CAG repeat ≥ 40 with a UHDRS-TMS ≥ five and a Total Functional Capacity score (TFC) ≥ 7. A burden of pathology score greater than 250 ((CAG repeat length − 35.5) × age) was required as a further inclusion criterion for the HD gene carrier group^2,10^. Healthy gene-negative partners (or family members in three instances) were recruited as controls (25 subjects). None of the participants suffered from a concomitant neurological or psychiatric disorder or had a history of severe head injury. The study was approved by the Medical Ethics Committee of the Leiden University Medical Center and written informed consent was obtained from all participants. All methods were performed in accordance with the relevant guidelines and regulations.

**Table 1:** Group characteristics and clinical scores.

	Healthy controls	Combined (pre)HD	preHD	Early HD
N	25	26	6	20
Gender male/female	7/18	10/16	1/5	9/11
Age in years, mean (SD)	52.7 (8.7)	49.7 (8.5)	49.1 (4.9)	49.9 (9.4)
Handedness R/L	24/1	22/4	5/1	17/3
Level of education (ISCED), median (range)	4 (6)	5 (5)	4.5 (4)	5 (5)
CAG repeat length, mean (SD)	n/a	43.2 (2.3)	41.3 (1.2)	43.8 (2.2)^¥^
Estimated years to onset, mean (SD)	n/a	n/a	10.8 (2.6)	n/a
Total functional capacity, mean (SD)	13.0 (0.2)	12.3 (1.2)*	12.8 (0.4)	12.1 (1.3)^Φ^
UHDRS-TMS, mean (SD)	1.3 (1.7)	10.5 (6.9)*	2.8 (2.1)	12.8 (6.1)^Φ^
SDMT, mean (SD)	54.7 (11.5)	49.3 (10.0)^	56.7 (10.4)	47.1 (9.0)*
SWR, mean (SD)	108.0 (16.1)	95.0 (14.5)*	99.0 (7.2)	93.9 (16.0)*
BDI-II, mean (SD)	3.6 (3.9)	6.6 (7.3)^	3.3 (2.9)	7.6 (8.0)^Φ^

N = number of participants, SD = Standard deviation, n/a = not applicable, ISCED = International Standard Classification of Education, CAG = Cytosine-Adenine-Guanine, UHDRS-TMS = Unified Huntington’s Disease Rating Scale-Total Motor Score, SDMT = Symbol Digit Modalities Test, SWR = Stroop Word Reading task, BDI-II = Beck Depression Inventory-II.

Significance at p ≤ 0.05 level: *significantly different from controls, ^Φ^significantly different from controls and preHD, ^¥^significantly different from preHD. ^p = 0.07.

### Clinical measures

The following clinical measures were evaluated in all participants: UHDRS-TMS, TFC, Symbol Digit Modalities Test (SDMT), Stroop Word Reading (SWR) and Beck Depression Inventory-II (BDI-II) scores.

The UHDRS-TMS is the current gold-standard which defines manifest disease state in HD. The SDMT and SWR have been shown to be sensitive neurocognitive measures in HD, independent of disease related motor effects^11^.

### EEG recording

The International 10–20 system was used for electrode placement using 19 Ag/AgCl electrodes. The average potential was used as a reference in subsequent analyses. Two horizontal bipolar eye movement leads and one for the electrocardiogram were applied to monitor artefacts. The EEG was recorded for three minutes with subjects at rest with eyes closed. Subjects were instructed to sit comfortably in a chair and close their eyes, but to remain awake. Subjects were alerted if they became visibly drowsy or if there were indications of that on the EEG. EEGs were recorded using a Nihon Kohden Neurofax 1200 system. Matlab (MathWorks® Version 7.1) and the LIBSVM toolbox^12^ were used for analyzing the data.

### EEG and statistical analysis

The analysis started by calculating the power spectrum followed by the connectivity and synchronization between electrodes. This was done to extract features from the recordings that reflect the variations of the spatial and temporal information in the multivariate data. First the power spectrum was calculated in the average montage for the signal at each individual electrode using a Fast Fourier Transformation (FFT) algorithm^13^ for consecutive 2 second segments with an overlap of 1 second. The EEG of each segment was subjected to a Bartlett window and a power spectrum using the FFT method was calculated, so for each electrode/lead N spectra were obtained, in which N was the number of segments. A final estimate for the power spectrum was then obtained by applying robust fits^14^ for each point in the spectrum, over the ensemble of N spectra. The second step of the analysis involved the connectivity and synchronization between electrodes, through the power spectrum of the auto correlation function between all possible pairs of electrodes. This was done in the average montage. The same segments were used as described above. The choice of 2 second segments resulted in a spectral resolution of 0.5 Hz. We chose to work with a spectral cut-off of 45 Hz. This resulted in 91 spectral power values for each spectrum. The total number of spectral estimates entering the evaluation was 19 for the spectra for each electrode as well as 171 for all the possible autocorrelation spectra. Together, there were 17290 spectral features for each qEEG. The full spectrum was considered for investigation of the group level differences between the single electrode spectra. For the statistical pattern recognition (SPR) analysis the feature set was reduced. To do so, each spectrum was first reduced by dividing it into overlapping bands of 8 Hz width with an overlap of 4 Hz. Each band was modulated by a Bartlett window reducing the number of features from 91 spectral features to 11. This procedure reduced the total number of features to 2090.

As the cohort in this study was small, it was important to avoid instability and overfitting in the SPR analysis if all features were taken into account simultaneously. This can occur even though support vector machine is applied in the SPR, which depend on the number of support vectors but not the number of features^15^. A subset of only 20 features were used in the analysis. The subset of features was chosen by applying a genetic algorithm that optimized the area under the curve (AUC) of the resulting receiver operator characteristic (ROC) curve^16^. The ROC statistics were estimated for each candidate feature subset using 10-fold cross-validation^17^. For comparison of bias, 3- and 5-fold cross-validations were also performed, where the resulting estimates of the ROC statistics did not differ significantly. The combined HD gene carrier group (26 subjects) was pooled in the EEG analysis due to low numbers of preHD participants when considered separately, where it was not feasible to create a separate classifier, and in order to increase overall power. Furthermore, combining data from the preHD group with the early HD group did not affect outcomes. A classifier was constructed that contrasted the control group and the HD gene carrier group. The classifier yielded an HD vs. control (HDvsCT) Index, ranging from 0 to 1, with low values for controls and high values indicating HD. The performance of the classifier was determined using repeated 10-fold cross-validation.

Correlations between the electrophysiology and clinical modalities were sought using a similar approach. In this case, however, principal component analysis (PCA) was applied on each feature subset. The linear Pearson correlation between the principal components and the clinical modalities was optimized. Statistical analysis of group demographics and clinical measures was performed using IBM SPSS Statistics (version 20, IBM, USA). Distributions and assumptions were checked and appropriate statistical tests were applied.

## Results

### Group characteristics and clinical scores

The groups did not differ significantly in terms of age, gender, handedness or level of education. TFC and SWR were significantly lower for the HD gene carrier group compared to the control group (p = 0.007 and p = 0.004, respectively; Mann–Whitney *U* test and independent-samples t-test, respectively). The HD gene carrier group had higher UHDRS-TMS than controls (p = 0.00001, independent-samples t-test). There was a trend for lower SDMT scores and higher BDI-II scores for the HD gene carrier group compared to controls (both p = 0.07; independent-samples t-tests). The early HD group had lower SDMT scores compared to controls only (p = 0.02; analysis of variance) and higher BDI-II scores compared to both preHD and controls (p = 0.04 and p = 0.01, respectively; analysis of variance). See Table 1 for a summary of these results.

### The HD classifier

**Figure 1:**
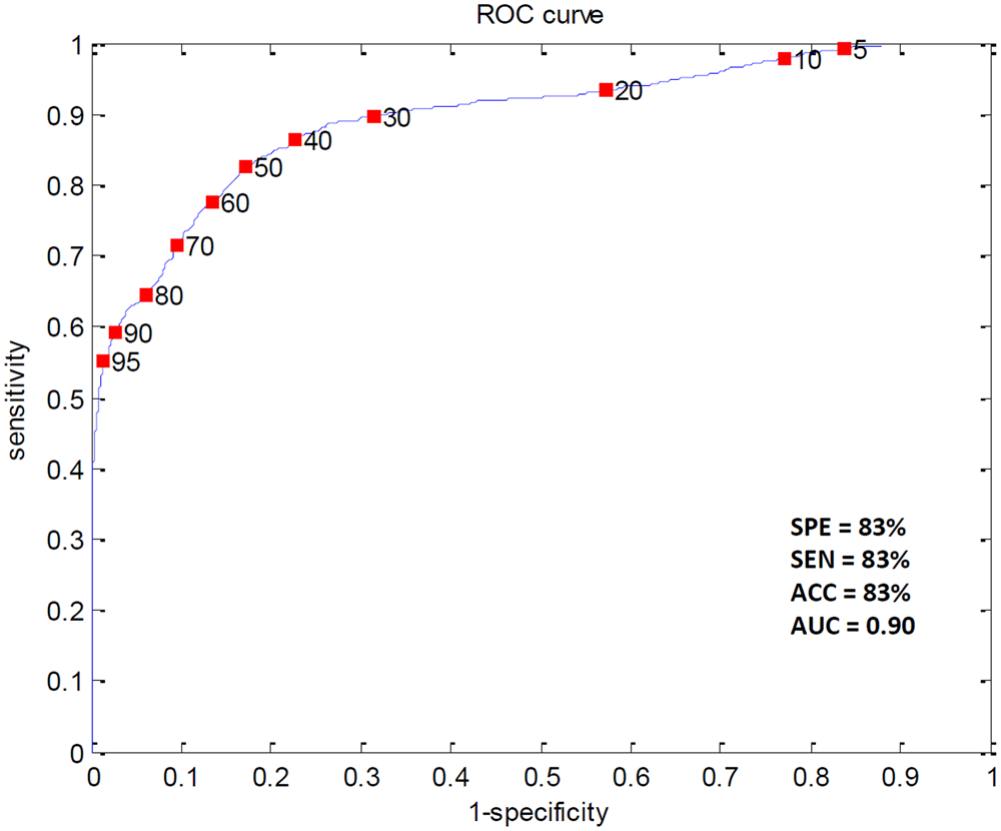
The ROC curve for the HD vs. control Index estimated with repeated 10-fold cross-validation along with the result. SPE = specificity; SEN = sensitivity; ACC = accuracy; AUC = area under the curve.

**Figure 2:**
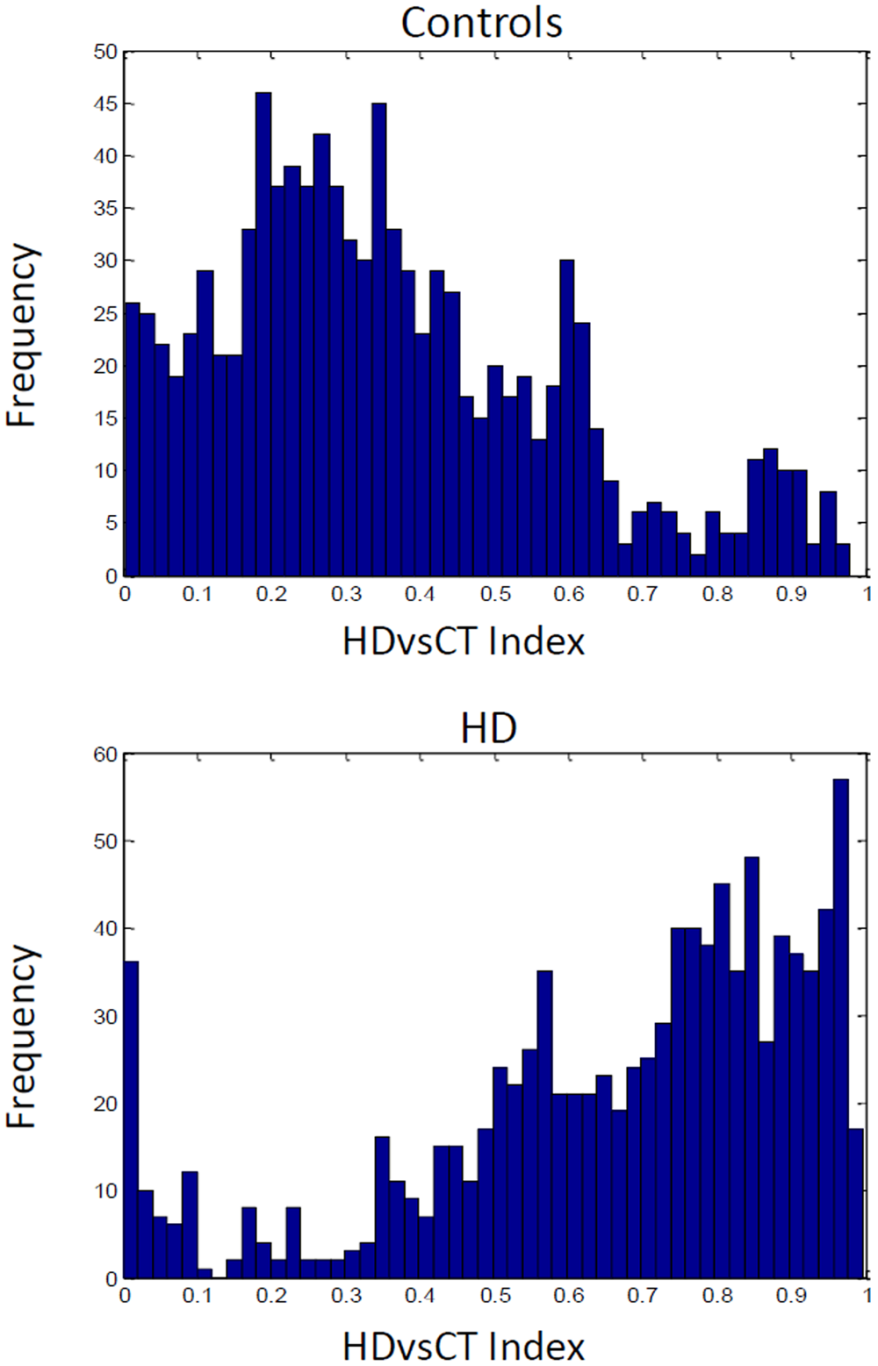
Controls and Huntington’s disease subjects in the HD vs. control (HDvsCT) Index as estimated with repeated 10-fold cross validation. The frequency is an estimate of the continuous likelihood distribution.

A classifier was constructed that optimized the contrast between the HD gene carrier and control groups with a specificity of 83%, a sensitivity of 83% and an accuracy of 83%. The AUC was 0.9 (Fig. 1). The estimated group distributions are illustrated in Fig. 2. There were no significant relationships between the HDvsCT Index and any of the clinical measures.

### Correlating qEEG subsets with clinical modalities

**Figure 3:**
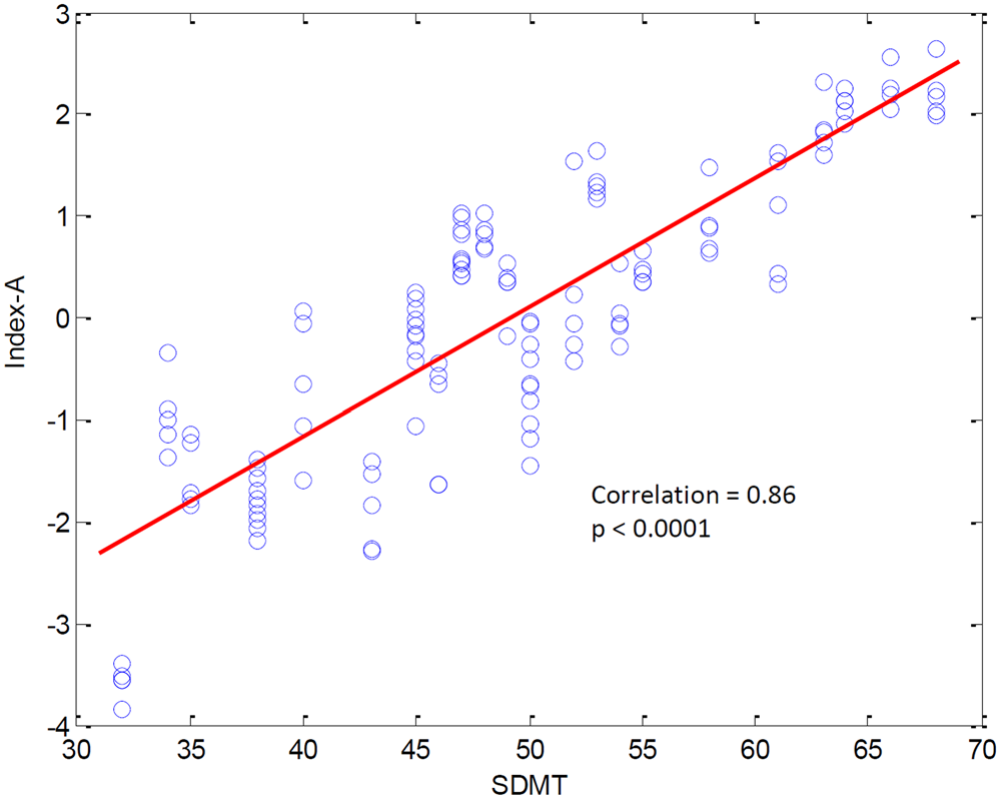
Relationship between Index-A and the SDMT score. The contribution of Index-A was evaluated in 5 consecutive segments of the EEG recording for each subject. All results are shown, illustrating the inter-subject variability of Index-A.

**Figure 4:**
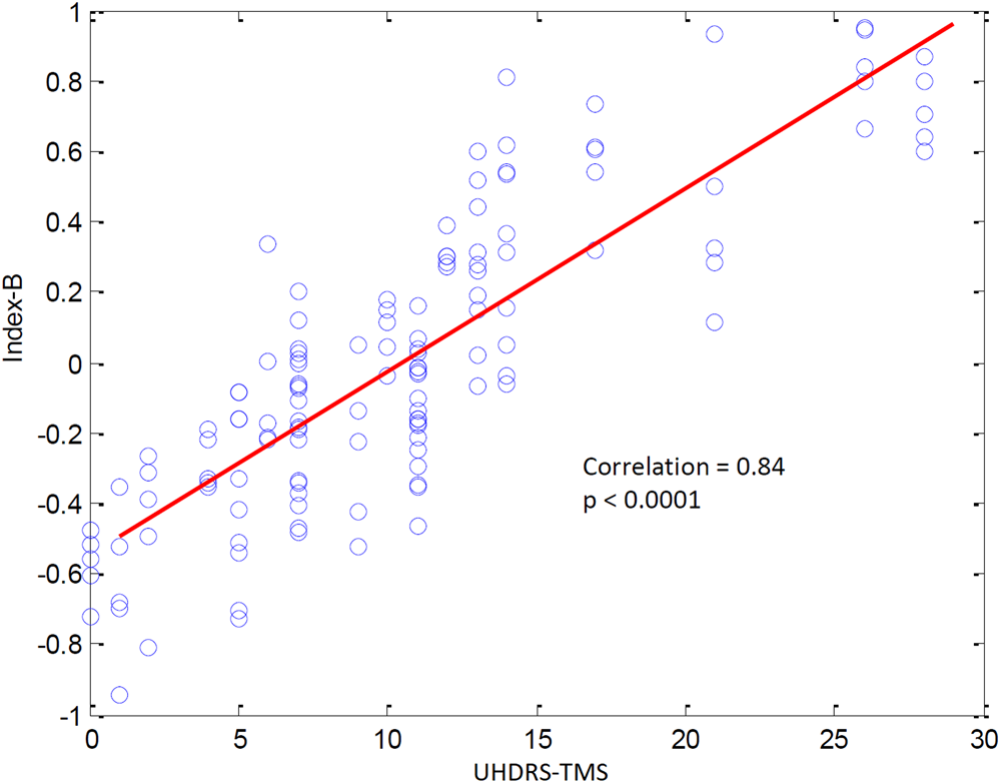
Relationship between Index-B and the UHDRS-Total Motor Score. The contribution of Index-B was evaluated in 5 consecutive segments of the EEG recording for each subject. All results are shown, illustrating the inter-subject variability of Index-B.

The analysis of the correlations between electrophysiological features and clinical modalities resulted in two highly significant correlations in the HD gene carrier cohort. The first factor, referred to as Index-A, correlated strongly with the SDMT score, see Fig. 3. Pearson’s correlation coefficient was 0.86 (p = 0.0001). The second factor, referred to as Index-B, correlated strongly with the UHDRS-TMS, see Fig. 4 (r = 0.84, p = 0.0001). See Supplementary Figs 1 and 2 for an overview of the spatial and spectral dependence of the coherences entering indices A and B.

### Full power spectrum analysis

The full power spectra for the 19 electrodes were evaluated and group averages were compared (Supplementary Fig. 3). The average spectra were significantly different (p = 0.001). Most prominently, the overall power was less in the HD gene carrier group. An extra resonance appeared in the average spectra of the HD gene carrier group at about 22 Hz, not present in the control group in the right temporal region. The alpha peak was distinctly divided into two peaks in the occipital, temporal and parietal areas.

### qEEG spectral differences

In the area of the anterior prefrontal cortex (Brodmann area 10; BA10), channels Fp1 and Fp2, the HD gene carrier group had a higher power than controls in the delta band. At all other locations significant difference in power was such that the power was higher in the control group except for the delta bands (higher in the HD gene carrier group): at the frontal eye fields (BA8), F3, F4 and Fz (theta); at the primary somatosensory cortex (BA2) and motor cortex (BA4), C3 (delta, theta and alpha), C4 (theta and alpha), Cz (theta); at the temporal regions influenced by the auditory somatosensory cortex (BA42), primary somatosensory cortex (BA2) and motor cortex (BA4), T3 (theta and alpha), T4 (delta, theta and alpha); and also influenced by the fusiform gyrus (BA37), T5 (theta and alpha), T6 (delta and theta); finally in the parietal area (BA7), Pz (theta and alpha). See Table 2 for a summary of these results, including p-values, t-statistics and Cohen’s d for effect sizes.

**Table 2:** Significant differences in qEEG spectral power.

Channel	Band	Power - Healthy controls (N = 25)	Power - Combined (pre)HD (N = 26)	*p*-value	t-statistic	Cohen’s *d*
Fp1	delta	4.5	4.9	0.004	−3.0	0.85
Fp2	delta	4.5	4.9	0.005	−2.9	0.82
F3	theta	3.3	3.0	0.007	2.8	−0.78
F4	theta	3.3	3.1	0.016	2.5	−0.70
Fz	theta	3.4	3.1	0.003	3.1	−0.88
C3	delta	3.6	3.8	0.048	−2.0	0.57
C3	theta	3.1	2.9	0.011	2.7	−0.74
C3	alpha	3.2	2.9	0.025	2.3	−0.65
C4	theta	3.1	2.9	0.004	3.0	−0.85
C4	alpha	3.2	2.9	0.022	2.4	−0.66
Cz	theta	3.3	3.1	0.024	2.3	−0.65
T3	theta	3.3	3.1	0.025	2.3	−0.65
T3	alpha	3.4	3.1	0.049	2.0	−0.56
T4	delta	3.9	4.1	0.031	−2.2	0.62
T4	theta	3.3	3.1	0.024	2.3	−0.66
T4	alpha	3.4	3.1	0.047	2.0	−0.57
T5	theta	3.5	3.2	0.009	2.7	−0.77
T5	alpha	3.7	3.4	0.025	2.3	−0.65
T6	delta	3.9	4.1	0.042	−2.1	0.58
T6	theta	3.4	3.2	0.042	1.8	−0.51
Pz	theta	3.2	3.1	0.031	2.2	−0.62
Pz	alpha	3.5	3.1	0.042	2.1	−0.58

Power values are log10-transformed. N = number of participants. Two-tailed t-test *p*-values are reported. Degrees of freedom = 49.

## Discussion

In this exploratory study, the qEEG automatic classification index proved to separate HD gene carriers from healthy controls with good specificity and sensitivity. This method has therefore a potential to be further developed as a biomarker in HD. The study also revealed strong correlations between qEEG features and the UHDRS-TMS and SDMT, both relevant clinical markers in HD research. Finally, global EEG average power spectra were shown to be significantly lower in the HD gene carrier group compared to controls and qEEG spectral differences between the groups were demonstrated.

Using the index created in this study, it is possible to separate EEGs of HD and control subjects with an accuracy of over 80%. Considering direct correlations between the index and commonly used clinical measures is interesting, though less likely to result in significant findings as the measure is derived globally from all recorded regions of the brain, therefore lacking specificity. The index did indeed not correlate with any of the commonly used clinical and neurocognitive measures in HD research. This finding is in line with a previous study using a classifier approach^9^. When specific EEG features were considered, highly significant correlations with the UHDRS and SDMT scores were found, disease measures that are known to be altered in a longitudinal fashion in the (pre-) manifest state compared to healthy controls. This highlights the importance of using different approaches in biomarker research based on structural and/or functional brain data. Analyses focusing on global versus local measures provide different insights on disease state and possible correlations with clinical measures. Previous machine learning studies using different MRI modalities to discriminate HD gene carriers and controls achieved accuracies up to 83% and 76%, respectively^18,19^, when specific regions affected by the disease were preselected for analysis.

On EEG average power spectra a global decrease in theta and alpha power in HD was found, while delta power was increased in a few brain areas in HD. As the earliest structural brain changes in HD start within the striatum, this conceivably leads to disrupted projections in the cortico-striato-thalamo-cortical loops, which in turn lead to disruptions in brain rhythms^20^. The striatum represents a crucial node in these loops^21^. Reductions in the theta band power in HD have been reported in previous studies^22–25^, while other studies found an increase in this band^9,26,27^. Reductions in the theta band power were correlated with increased cognitive and motor deficits^23^. There seems to be consensus in the literature regarding globalized reductions in the alpha band in (pre)HD^9,23,24,27–29^. Some studies reported that reductions in the alpha band correlated significantly with increases in cognitive and motor deficits in HD^22,23^, while others could not replicate this finding^9^. Both theta and alpha EEG rhythms appear to reflect important neuronal processes in human cognition^30–32^. Deacreases^23,27^, as well as increases^22^ in beta power in HD have been reported, something we could not replicate. Most studies point to an increase in delta power in HD^9,22,23,25,27,29^, which is corroborated by findings in our study. It has been observed that alterations in delta power might be disease stage dependent and increase in advanced stages of HD^20^. This might explain the localized differences in delta power between the groups observed in this particular study sample, which represents premanifest or early stage patients.

The GABAergic network is postulated to be a driving force in producing synchronized brain oscillations^33^. Combined with the knowledge that dysfunction and loss of GABAergic neurons occurs early on in the striatum of HD^34,35^ we hypothesize that the difference found in this study, both in the classification index as well as in differences in power spectra, are primarily derived by a deregulation of brain network oscillations through GABAergic dysfunction in HD. Another potential explanation for these findings might be a neurodevelopmental difference of HD brains reflecting an endophenotype. To explore the latter point, it is necessary to conduct longitudinal trials evaluating the potential progressive nature of these differences with advancing disease.

In this study we have observed several statistically significant results in the performance of classifiers as well as indices designed to correlate with relevant modalities related to HD progression. As with EEG related physiological interpretation in general, it is very hard to assign physiological meaning to these indices as the knowledge of relationships between EEG activity and the underlying physiology are poorly known or understood. The field is still in its data driven empirical era, which the present work contributes to. We have also observed significant differences between classical qEEG features when comparing between HD gene carriers and controls. These are exploratory findings limited in scope when it comes to the number of subjects participating. It is therefore pertinent to confirm these findings in independent studies conducted with pre-defined end points. Also, there is an increased risk of overfitting the separation model when using a small sample size as the one in this study. Another potential limitation is the use of the same system to record all EEGs, possibly reducing the validity of the model on other EEG equipment. Also, as this is a cross-sectional study, we can only speculate about the expected changes to the findings occurring during clinical deterioration in HD. Therefore, longitudinal studies are needed to evaluate the true usefulness of these indices. However, the fact that we have found indices strongly correlating with clinical markers of decline support the notion of a measurable progressive change in HD brain function rather than a purely neurodevelopmental difference.

## Conclusion

In this exploratory study we show promising results where qEEG related modalities may help to unravel how HD evolves and how different areas of the brain are influenced as the condition progresses. The indices correlating with modalities changing with the progression of the disease may lead to tools based on qEEG that can help monitor efficacy in intervention studies. These points will need further independent studies before such applications can be put into force.

## Electronic supplementary material

Supplementary Information

## Data Availability

The datasets generated during the current study are available from the corresponding author on reasonable request.
