# Supplementary material for: EEG may serve as a biomarker in Huntington’s disease using machine learning automatic classification
Source: Sci Rep. 2018 Oct 31;8:16090. doi: 10.1038/s41598-018-34269-y (PMC6208376; doi:10.1038/s41598-018-34269-y)
Supplement: Supplementary file 1 — Supplementary Information [file 41598_2018_34269_MOESM1_ESM.docx]

EEG may serve as a biomarker in Huntington’s disease using machine learning automatic classification

Omar F.F. Odish^1*^, Kristinn Johnsen^2^, Paul van Someren^3^, Raymund A.C. Roos^3^, J. Gert van Dijk^3^

^1^ Department of Neurology, University Medical Center Groningen, Groningen, The Netherlands

^2^ MentisCura ehf., Reykjavík, Iceland

^3^ Department of Neurology, Leiden University Medical Center, Leiden, The Netherlands

* Corresponding author at: Department of Neurology, University Medical Center Groningen, P.O. Box 30.001, 9700 RB Groningen, The Netherlands. Tel: +31 50 361 2401 (secretary); fax: +31 50 361 1707. E-mail address: o.f.f.odish@umcg.nl

### Supplementary Figures

### Supplementary Figure 1

###
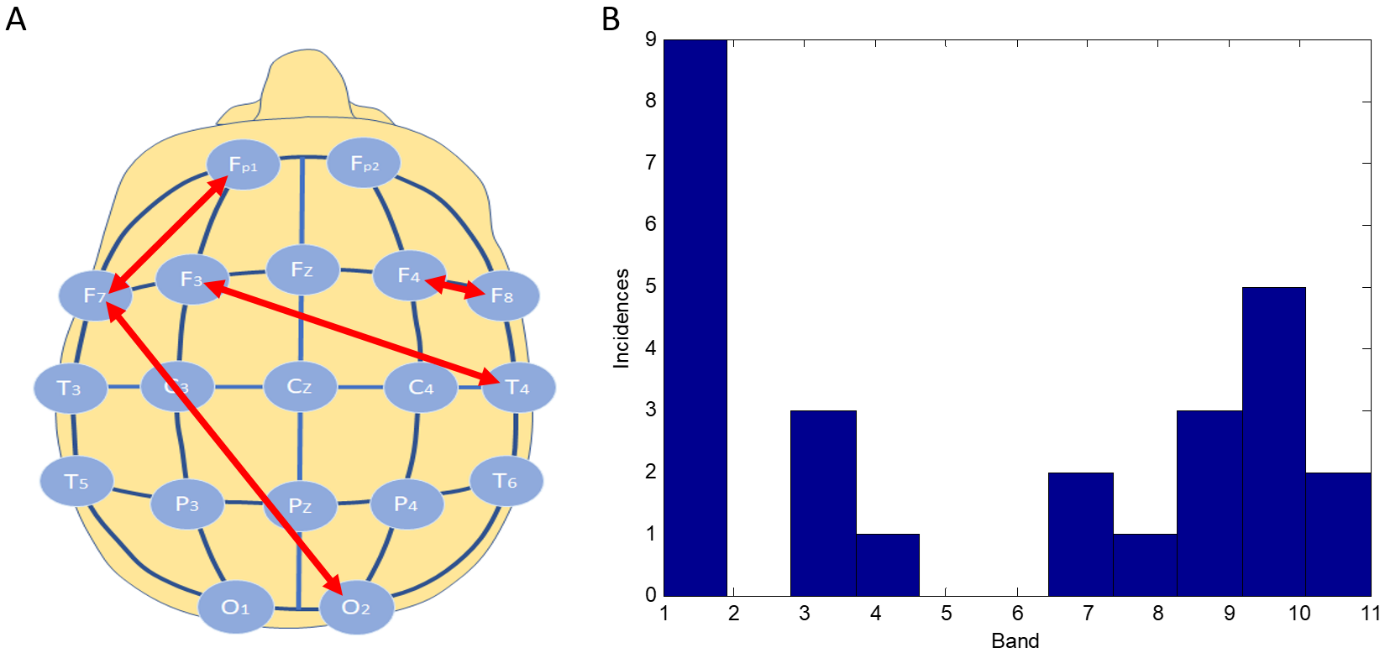


### Spatial (A) and spectral (B) dependence of the coherences entering Index-A.

### Supplementary Figure 2

###
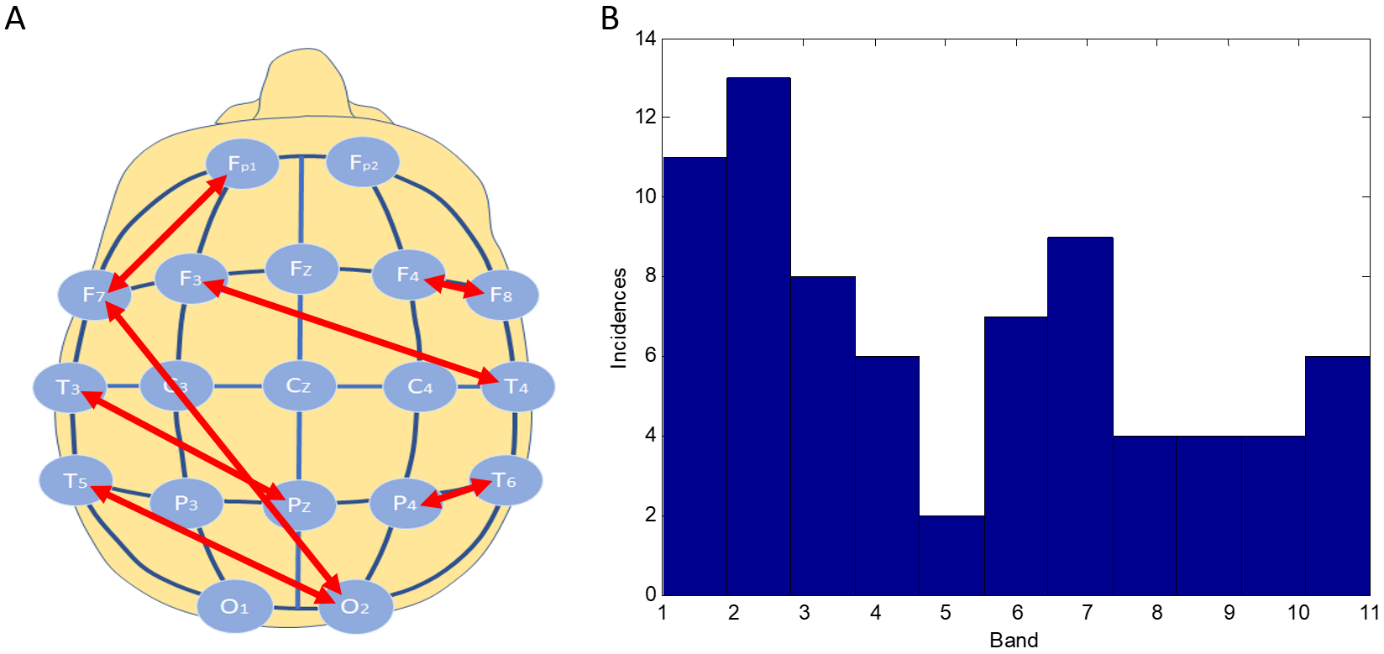


### Spatial (A) and spectral (B) dependence of the coherences entering Index-B.

### Supplementary Figure 3

###
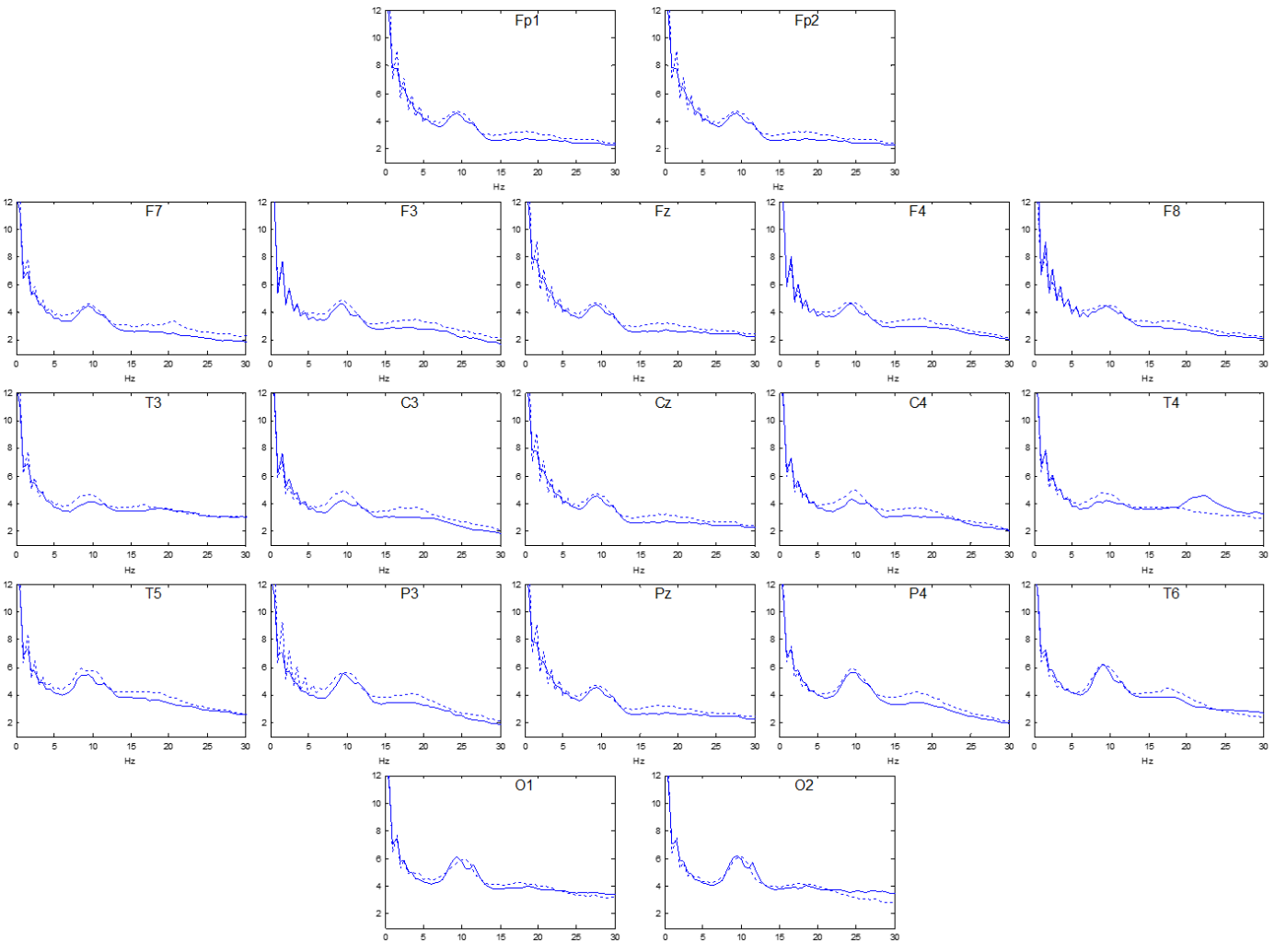


### The average full power spectra on group level. The dotted curves are the average over the control group. The solid curves are the average over the HD gene carrier group.
